# The Metathoracic Scent Gland of the Leaf-Footed Bug, *Leptoglossus zonatus*

**DOI:** 10.1673/031.013.14901

**Published:** 2013-12-11

**Authors:** J. Gonzaga-Segura, J. Valdez-Carrasco, V. R. Castrejón-Gómez

**Affiliations:** 1 Becario COFAA. Laboratorio de Ecología Química de Insectos. Departamento de Interacciones Planta-Insecto. Centro de Desarrollo de Productos Bióticos del Instituto Politécnico Nacional. Carretera Yautepec, Jojutla, Km. 6 Calle CEPROBI No. 8, Col. San Isidro, Yautepec, Morelos, Mexico, C.P. 62731; 2 Laboratorio de Morfología de Insectos. Colegio de Posgraduados en Ciencias Agrícolas Campus Montecillo. Carretera México-Texcoco km 36.5, Montecillo, Texcoco, Estado de México, C.P. 56230

**Keywords:** behavior, Heteroptera, morphology, pest, semiochemicals

## Abstract

The metathoracic scent gland of 25-day-old adults of both sexes of the leaf-footed bug, *Leptoglossus zonatus* (Dallas) (Heteroptera: Coreidae), are described based on optical microscopy analysis. No sexual dimorphism was observed in the glandular composition of this species. The gland is located in the anteroventral corner of the metathoracic pleura between the middle and posterior coxal pits. The opening to the outside of the gland is very wide and permanently open as it lacks a protective membrane. In the internal part, there is a pair of metathoracic glands that consist of piles of intertwined and occasionally bifurcated cellular tubes or columns. These glands discharge their pheromonal contents into the reservoir through a narrow cuticular tube. The reservoir connects with the vestibule via two opposite and assembled cuticular folds that can separate muscularly in order to allow the flow of liquid away from the insect. The external part consists of an ostiole from which the pheromone is emitted. The ostiole is surrounded by a peritreme, a structure that aids optimum pheromone dispersion. The described gland is of the omphalien type.

## Introduction

The leaf-footed bug, *Leptoglossus zonatus* (Dallas) (Heteroptera: Coreidae), has a distribution range that extends from the northern United States to southern Argentina (Allen 1969; Matrangolo and Waquil 1994). This pest feeds on crops such as corn, sorghum, melon, avocado, pomegranate, guava, sesame, sunflower, potato, tomato, beans, and some ornamental plants (Kubo and Batista 1992; Jones 1993; Matrangolo and Waquil 1994; Raga et al. 1995; Pillegi de Souza and Do Amaral Filho 1999; Xiao and Fadamiro 2010). Furthermore, it is one of the most frequent pests in plantations of *Jatropha curcas*, which is grown for the production of biodiesel (Morales et al. 2011). Both nymphs and adults cause direct damage, feeding on leaves and fruit, provoking fruit abortion and seed malformation (Matrangolo and Waquil 1994; Grimm and Fuhrer 1998; Xiao and Fadamiro 2010).

The nymphs and adults of various true bug families are characterized by the development of odoriferous glands that release irritating substances known as alarm pheromones (Farine et al. 1992, 1993; Leal et al. 1994; Steinbauer and Davier 1995; Blatt et al. 1998; Ho and Millar 2001). Nymphs of various Heteroptera families secrete alarm pheromones via the dorsal abdominal glands. Some examples include the Coreidae *Carlisis wahlbergi* Stal (De Lange and Rensburg 1982), *Megalotomus quinquespinosus* Say, *Alydus eurinus* Say, *A. pilosulus* Herrich-Schaeffer, *Leptoglossus clypealis* Heidemann, *L. oppositus* Say, *Archimerus alternatus* Say, and *Acanthocerus (Euthochtha) galeator* Fabricius (Aldrich and Yonke 1975) and the Pentatomidae, *Nezara viridula* (L.) (Pavis et al. 1994). However, adults secrete alarm pheromones through the metathoracic scent glands (MTGs) (Staddon 1979; Aldrich 1988). Alarm pheromones produced by various Heteroptera species have been identified in the Coreidae *Cor eus marginatus* L. (Durak and Kalender 2007b), *L. occidentalis* Heidemann (Blatt et al. 1998), *L. phyllopus* L. (Aldrich et al. 1978), *Holopterna allata* W. (Staddon et al. 1979), *Carlisis wahlbergi* Stal (De Lange and Rensburg 1982), and *Merocoris distinctus* Dallas (Aldrich and Yonke 1975), the Scutelleridae *Eurygaster Maura L.* (Durak and Kalender 2007a) and *E. integriceps* (Puton) (Hassani et al. 2010), the Pentatomidae *Graphosoma lineatum L.* (Durak and Kalender 2008) and *Erthesina fullo* T. (Kou et al. 1989), and the Alydidae *Riptortus clavatus* (Thunberg) (Leal and Kadozawa 1992). The MTGs also secrete substances with other purposes such as mating, intraspecific aggregation, and repellency (Regnier and Law 1968; Aldrich et al. 1979; Staddon 1979; Staddon 1986; Gunawardena and Bandumathie 1993).

Some species of Coreidae such as *L. occidentalis* and *L. zonatus* are easily perturbed and emit defensive substances to combat their natural enemies that also act as alarm volatiles between conspecifics (Blatt et al. 1998). Leal et al. (1994) and subsequently Blatt et al. (1998) identified the components of the alarm pheromone of *L. zonatus.* These compounds are secreted by the MTG. However, these authors did not describe this or any other gland in *L. zonatus* adults. There are few studies in which the MTGs have been described, such as that by Durak and Kalender (2007b) for *C. marginatus* and the excellent description by Hepburn and Yonke (1971) of the MTGs in various species of three families belonging to Coreoidea: Coreidae, Alydidae, and Rhopalidae. The aim of this study was to generate a morphological description of the MTGs in adult male and female *L. zonatus.*

## Materials and Methods

A total of 25 one-day-old adults of both sexes were obtained from a breeding colony maintained in the Centro de Desarrollo de Productos Bióticos (Center for Development of Biotic Products) and were preserved in 70% ethanol. The thorax and the first abdominal segments were separated by transverse cuts and subsequently digested in 10% potassium hydroxide at 80° C for 20 min. The samples were washed in running water, and the digestion was stopped using acidulated water (1% acetic acid). The microscopic parts of these samples were dehydrated in absolute ethanol, rinsed in xylol, and mounted in Canada balsam.

The *in situ* study of the glands and related organs was carried out using anaesthetized specimens in a CO_2_ atmosphere, and dissection was conducted when the specimens were submerged in running water.

Insects fixed in Duboscq-Brazil for two weeks were used for the study of the internal organs and their musculature. The specimens were washed and preserved in 70% ethanol until used for observations. Under an optical microscope, a longitudinal cut was performed separating the thorax, and the muscular tissue was removed until only the MTG remained.

Optical microscopy and photography for subsequent image description was conducted using a Carl Zeiss Tessovar microscope and a Photomicroscope III (Carl Zeiss, www.zeiss.com) with a PAXcam 5 digital camera for microscopes (PAXcam, www.paxcam.com). The GIMP 2.6.11 program (www.gimp.org) was used for editing digital images.

## Results

**Figure 1.**
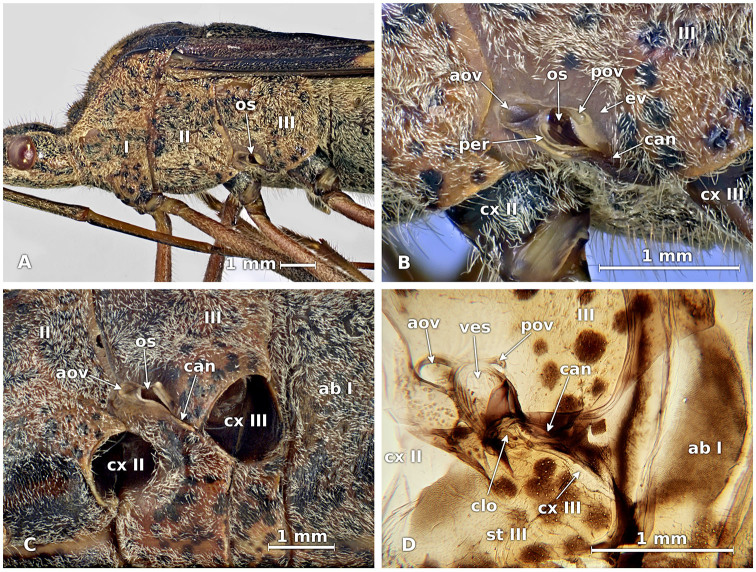
External morphology and location of the metathoracic scent gland in *Leptoglossus zonatus.* A, location of ostiole. B, ostiole and associated structures. C, canal from gland to metathoracic coxal pit. D, internal view of vestibule and canal. I, II, III, prothoracic, mesothoracic, and metathoracic pleura, respectively; ab I, first abdominal plate; aov, anterior oval; can, canal from ostiole to coxal pit III; clo, closing apparatus; cx II, cx III, mesothoracic and metathoracic coxa, respectively, or coxal pits; ev, evaporative surface; os, ostiole; per, peritreme; pov, posterior oval; st III, metathoracic sternum; ves, vestibule. Scale bars: mm. High quality figures are available online.

The openings of the MTGs are located in the anteroventral corners of the metathoracic pleura on each side above the middle coxal pit (Figure 1A). The ostiole of these glands is very wide and permanently open and lacks a protective membrane. The edge of the ostiole is surrounded by a yellowish cuticle and is clearer than the thoracic plate. It forms a peritreme on the edge of the ostiole with an oval situated in the translucent cuticle of the anterior part of the orifice. The oval is highly convex, with a raindrop-shaped outline and the apex at the back (Figure 1B).

The same peritreme forms two edges, or lips, that flank the ostiole: one anterior, the other posterior, above which there is another convex oval. The edge of the ostiole (evaporative surface) is rounded on the anterior part but elongated towards the posterior part where it continues as a deep canal that stretches in a diagonal line downward and backward, reaching the metathoracic coxal pit (Figure 1C).

**Figure 2.**
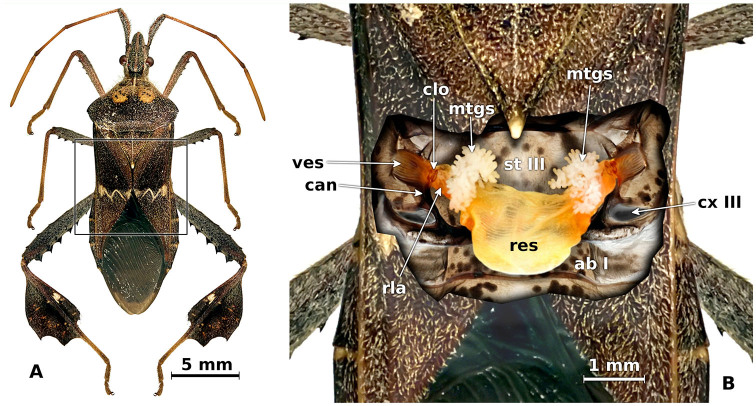
Glandular complex in the metathorax of *Leptoglossus zonatus.* A, dorsal view of a female showing the area enlarged in B. B, metathoracic scent glands and associated structures inside the metathorax. ab I, first abdominal plate; can, canal from ostiole to coxal pit III; clo, closing apparatus; cx III, metathoracic coxa; mtgs, metathoracic scent glands; res, reservoir; rla, reservoir lateral arms; st III, metathoracic sternum; ves, vestibule. High quality figures are available online.

The ostiole is a hollow cuticle that enters the inside of the metathorax, forming a dorsoventrally flattened tubular conduct with distinct longitudinal grooves on the inside (Figure 1D). This cuticular chamber is called the vestibule, and its base connects to the lateral mouth of the glandular reservoir. Here the lateral edges of the reservoir are present and consist of a large sac of translucent membranous cuticle that in fresh insects is characterized by an intense orange color and extends behind and over the metathoracic sternum to the second abdominal sternum (Figure 2).

The reservoir membrane is completely lined with transverse folds that are distributed uniformly over the entire organ. The reservoir observed in both sexes is a bright orange sac. The MTGs, which store their secretions in the reservoir, run into the anterior edges of the reservoir lateral arms by means of a narrow, dark cuticle tube. The glands are piled or intertwined tubes and occasionally bifurcated cellular columns that rest on top of the lateral extensions of the reservoir (Figure 2). No lateral glands were observed growing in the reservoir wall.

**Figure 3.**
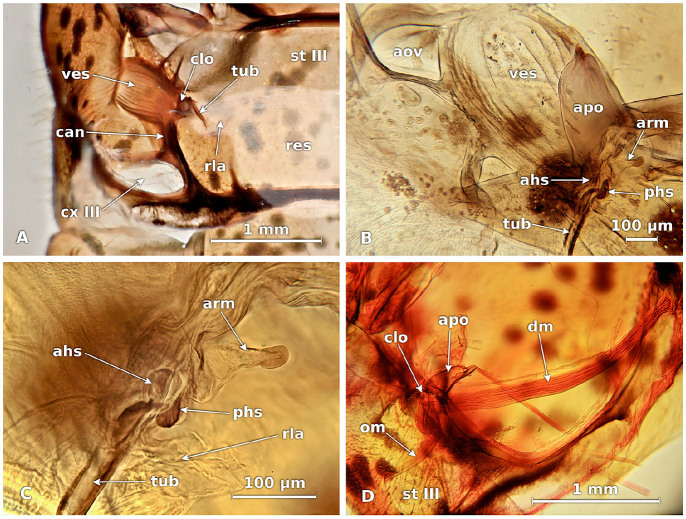
Closing apparatus of the metathoracic scent gland of *Leptoglossus zonatus.* A, dorsal view of the left vestibule. B, inner view of vestibule and closing apparatus. C, sclerites of the closing apparatus. D, dilator and oclussal muscles, ahs, anterior hemispheric sclerite; aov, anterior oval; apo, pleural apophysis; arm, conic arm; can, canal from ostiole to coxal pit III; clo, closing apparatus; cx III, metathoracic coxal pit; dm, dilator muscle; om, oclussal muscle; phs, posterior hemispheric sclerite; res, reservoir; rla, reservoir lateral arms; st III, metathoracic sternum; tub, tube from glands to reservoir; ves, vestibule. High quality figures are available online.

The vestibule (Figure 3A, B) is connected to the reservoir by two opposite cuticular folds arranged in such a way that they can be separated by muscular action to allow the flow of liquid to the exterior. This closing apparatus (Figure 3B, C) possesses an anterior hemispheric sclerite with a flattened part joined to its equivalent on the opposite fold.

The posterior hemispheric sclerite forms a conic arm that extends backward and has a round and flattened end. Both folds can be separated to open the conduct through the action of a pleural dilator muscle (Figure 3D) that has an elongated and fine structure. This muscle follows a curved path that begins at the anterior dorsal margin of the metathoracic pleura, and it possesses two branches at its insertion. The thinnest branch is implanted in the posterior hemispheric sclerite, and the rest of the muscle is inserted in the conic arm. The anterior hemispheric sclerite moves backwards and is pulled by a short, curved oclussal muscle (Figure 3B) that is attached to the sides of the metathoracic sternum.

## Discussion

Hepburn and Yonke (1971) described the MTGs for several species of *Leptoglossus.* However, *L. zonatus* was not included in this study. At a later date, the alarm pheromone of this species was identified, but the MTG was not described (Leal et al. 1994; Blatt et al. 1998). In male and female *L. zonatus*, the MTG is located in a ventral position in the posterior part of the metathorax, as reported for different species of Heteroptera by Staddon (1979) and Hepburn and Yonke (1971). In *Graphosoma lineatum* (Pentatomidae), this gland was reported to be found between the 2^nd^ and 3^rd^ coxa (Durak and Kalender 2008). This type of gland was also found in *Eurygaster maura* (Scutelleridae), *Coreus marginatus* (Coreidae) (Durak and Kalender 2007a, b), and *Dolycoris baccarum* (Pentatomidae) (Durak 2008) and is described as a volatile-producing gland located between the mesothorax and the metacoxa that is composed of a reservoir and a pair of lateral glands connected to the reservoir by a conduct.

The gland of *L. zonatus* described in this study was characterized by just one ostiole, which according to the classification presented by Carayon (1971), is considered to be of the omphalien type. In other species, this gland consists of two ostioles and is classified as the diastomien type (Carayon 1971). Some examples of this type of gland have been cited in *E. Maura, C. marginatus, G. lineatum* (Durak and Kalender 2007a, b; 2008), and *D. baccarum* (Durak 2008). However, the function attributed to these types of glands has not been established. Only a phylogenetic analysis has been conducted, as the species that have the omphalien type of gland are more primitive than those that do not.

Carayon (1971), Durak and Kalender (2007a, b; 2008), Durak (2008), and Carver (1990) mentioned that the evaporative surface or peritreme, a structure associated with the MTGs, is present in different species of Heteroptera. The Peritreme is formed by mushroom-shaped structures whose principal function is to improve pheromone dispersal upon movements of the insect's coxa (Carayon 1971; Hepburn and Yonke 1971; Durak and Kalender 2007a, b). This has also been observed in species belonging to the Reduviidae family and in *L. clypealis* (Wang and Millar 2000; Weirauch 2006). However, other authors suggested that the main purpose of this structure is to prevent secretions from flowing to the rest of the body, especially the tracheal openings (Remold 1963).

As reported in the majority of heteropterans (Staddon 1979), the reservoir described in *L. zonatus* is not associated with any other muscle. The content of this structure is possibly excreted as a result of an increase in hemolymph pressure caused by abdominal muscle movements. No lateral glands were observed in the reservoir of *L. zonatus*, in contrast to other heteropterans (Staddon 1979). However, the lateral extremes of the reservoir or secretory tubules of *L. zonatus* are articulated by the dorso-ventral muscles. Similar results were reported for *Oncopeltus fasciatus* (Dallas) (Heteroptera: Lygaeidae (Johansson 1957). No accessory or secondary glands were observed in *L. zonatus.* Various studies have shown that the middle reservoir accessory gland secretes aldehydes and alcohols (the characteristic true bug smell), while the lateral glands secrete esters (a pleasant smell) (Johansson 1957; Games and Staddon 1973; Aldrich 1978). As in this study, Waterhouse and Gilby (1964) and Staddon (1979) drew attention to the intense orange pigmentation of the reservoir. The first group suggested that the color intensity could be a signal to prevent autotoxicity; however, the composition and function of this structure have not been studied in detail.

The morphological characteristics of the different MTG structures, such as the ostiole, the peritreme, and the evaporative surface, can all be used for taxonomic, systematic, and phylogeny purposes in Heteroptera (Carayon 1971; Hepburn and Yonke 1971; Kamaluddin and Ahmad 1988; Eger and Baranowski 2002; Durak and Kalender 2007a, b; 2008).

The MTG in Heteroptera plays an important role in intra- and interspecific chemical communication. In this study, the previously uncharacterized MTG of *L. zonatus* adults was described. This knowledge may contribute to a better understanding of the chemical ecology of this species; however, the functionality of this organ has not been completely clarified, and to achieve this, *L. zonatus* chemical ecological, biochemical, histological, and behavioral studies need to be implemented.

## Abbreviations

MTG,: metathoracic scent gland
